# Transition und Transfer von der Pädiatrie in die Erwachsenenmedizin

**DOI:** 10.1007/s10354-026-01140-z

**Published:** 2026-03-18

**Authors:** Valentina Obermayr, Ingrid Pichler, Dorothea Appelt, Matthias Baumann, Elisabeth Binder, Ralf Geiger, Klaus Kapelari, Thomas Müller, Evelyn Rabensteiner, Michaela Sailer-Höck, Siegfried Waldegger, Anna Zschocke, Sabine Scholl-Bürgi, Daniela Karall

**Affiliations:** 1 Klinik für Pädiatrie, Angeborene Stoffwechselstörungen, Medizinische Universität Innsbruck, Anichstraße 35, 6020 Innsbruck, Österreich; 2 Bereichsverwaltung, Tirol Kliniken, Landeskliniken Innsbruck, Innsbruck, Österreich; 3 Klinik für Pädiatrie III, Cystische Fibrose, Medizinische Universität Innsbruck, Innsbruck, Österreich; 4 Klinik für Pädiatrie I, Neuropädiatrie, Medizinische Universität Innsbruck, Innsbruck, Österreich; 5 Klinik für Pädiatrie I, Diabetologie, Medizinische Universität Innsbruck, Innsbruck, Österreich; 6 Klinik für Pädiatrie III, Kardiologie, Medizinische Universität Innsbruck, Innsbruck, Österreich; 7 Klinik für Pädiatrie I, Endokrinologie, Medizinische Universität Innsbruck, Innsbruck, Österreich; 8 Klinik für Pädiatrie I, Gastroenterologie und Hepatologie, Medizinische Universität Innsbruck, Innsbruck, Österreich; 9 Klinik für Pädiatrie I, Hämatologie und Onkologie, Medizinische Universität Innsbruck, Innsbruck, Österreich; 10 Klinik für Pädiatrie I, Rheumatologie, Medizinische Universität Innsbruck, Innsbruck, Österreich; 11 Klinik für Pädiatrie I, Nephrologie, Medizinische Universität Innsbruck, Innsbruck, Österreich; 12 Klinik für Pädiatrie III, Pneumologie und Allergologie, Medizinische Universität Innsbruck, Innsbruck, Österreich

**Keywords:** Transition, Pädiatrie, Erwachsenenmedizin, Chronische und Seltene Erkrankungen, Transition, Pediatrics, Adult Medicine, Chronic and Rare Diseases

## Abstract

**Einleitung:**

Durch verbesserte Diagnostik und Therapie erreichen mehr Kinder und Jugendliche mit chronischen und seltenen Erkrankungen das Erwachsenenalter. Infolgedessen steigt der Bedarf an Erwachsenen-medizinischer weiterer Betreuung dieser Patientinnen und Patienten. In der vorliegenden Erhebung wurden die Patientinnen und Patienten an der Universitätsklinik Innsbruck für Pädiatrie (Bereiche: Gastroenterologie und Hepatologie, Hämatologie und Onkologie, Nephrologie, Endokrinologie, Diabetologie, Rheumatologie, Neuropädiatrie, angeborene Stoffwechselstörungen, Pneumologie und Allergologie, Kardiologie und Cystische Fibrose) erfasst, die einer Transition bedürfen, als Grundlage für das Verbesserungspotenzial an der Schnittstelle zwischen Pädiatrie und Erwachsenenmedizin.

**Methoden:**

Als Querschnittserfassung wurde die Anzahl der Patientinnen und Patienten der pädiatrischen Spezialbereiche für das Jahr 2023 erfasst. Die Gesamtanzahl (*n* = 12.078) wurde eingeteilt in unter 16 Jahre (*n* = 9635), zwischen 16 und 18 Jahren (*n* = 1288) und über 18 Jahre (*n* = 1155). Zu den Herausforderungen der Transition wurden halbstrukturierte Interviews mit den Leitern und Leiterinnen der pädiatrischen Spezialbereiche bzw. mit einer Stellvertreterin oder einem Stellvertreter geführt.

**Ergebnisse:**

Von den 12.078 Betroffenen waren im Bereich Angeborene Stoffwechselstörungen 21,83 % Patientinnen und Patienten und im Bereich Kardiologie (angeborene Herzfehler) 14,01 % Patientinnen und Patienten über 18 Jahre alt. In den Bereichen Cystische Fibrose und Hämatologie und Onkologie lag der Anteil der über 18-Jährigen bei 53,87 % bzw. 19,67 %, wobei in diesen beiden Bereichen die Patientinnen und Patienten vereinbarungsgemäß in pädiatrischer Betreuung verbleiben und die Betreuung dieser Patientinnen und Patienten einem klar definierten Transitionsprozess an der Klinik unterliegt. In den restlichen Bereichen wurde der Transitionsprozess bereits vollzogen. Die Interviews bestätigten die vorliegenden Zahlen, indem sie die Transition auf dem jeweiligen Stand beschrieben.

**Schlussfolgerung:**

Zusammenfassend erfolgt die Transition bereits in einem Großteil der pädiatrischen Spezialbereiche. Ausbaupotenzial bieten die Bereiche Angeborene Stoffwechselstörungen und Kardiologie, während die Bereiche Cystische Fibrose und Hämatologie und Onkologie einem eigenen Transitionsmodell folgen.

## Abstract (en)

**Aim:**

With improved diagnostics and therapy, more children and adolescents with chronic and rare diseases are reaching adulthood. As a result, the need for adult medicine specialists for the continued care of these patients is increasing. In this survey, the patients at the University Hospital Innsbruck for Pediatrics (departments: Gastroenterology and Hepatology, Hematology and Oncology, Nephrology, Endocrinology, Diabetology, Rheumatology, Neuropediatrics, Inherited metabolic Disorders, Pulmonology and Allergology, Cardiology, and Cystic Fibrosis) who are due for transition were recorded. This survey was intended to serve as a basis for identifying potential improvements at the interface between pediatrics and adult medicine.

**Methods:**

As a cross-sectional survey, the number of patients in the pediatric specialty departments for the year 2023 was recorded. The total number (*n* = 12,078) was divided into under 16 years of age (*n* = 9635), between 16 and 18 years of age (*n* = 1288), and over 18 years of age (*n* = 1155). To explore the challenges of transition, semi-structured interviews were conducted with the heads of the pediatric specialty areas or with a deputy.

**Results:**

Of the 12,078 patients, 21.83% of patients in the field of inherited metabolic disorders and 14.01% of patients in the field of cardiology (congenital heart defects) were over 18 years of age. In the fields of cystic fibrosis and hematology and oncology, the proportion of patients over 18 years of age was 53.87% and 19.67%, respectively. In these two fields, patients remain under pediatric care by agreement, and the care of these patients is subject to a clearly defined transition process within the clinic. In the remaining departments, a completed transition process can be observed. The interviews confirmed the available figures by describing the status of the transition in each department.

**Conclusion:**

In summary, the transition is already taking place in the majority of pediatric specialty departments. There is potential for further development in the fields of inherited metabolic disorders and cardiology, while the fields of cystic fibrosis and hematology and oncology have their own transition model.

Die Adoleszenz und der Übergang ins Erwachsenenalter sind eine besonders sensible Phase im Verlauf des Lebens, die durch zahlreiche entwicklungsbedingte und individuelle Veränderungen geprägt ist. Diese Transition stellt bereits für gesunde Jugendliche eine komplexe und herausfordernde Lebensphase dar, doch Jugendliche mit chronischen und seltenen Erkrankungen sehen sich zusätzlich mit weiteren spezifischen Hürden und Herausforderungen konfrontiert [1, 2]. Diese Studie beschreibt den Transitionsprozess in den jeweiligen Spezialbereichen der Pädiatrie I (Allgemeine Pädiatrie) und III (Kardiologie und Pulmonologie) der Universitätsklinik Innsbruck.

## Einleitung

Der Wechsel von Patientinnen und Patienten mit chronischen Erkrankungen von der Kinder- und Jugendmedizin in die Erwachsenenmedizin ist bislang nicht standardisiert. Er wird mit dem Ausdruck „Transition“ beschrieben. Dabei werden den zuständigen Erwachsenenmedizinerinnen und Erwachsenenmedizinern die Informationen über Betroffene und deren Zustand übergeben. Zum Teil impliziert der Begriff „Transition“ auch einen „Transfer“, also den Ortswechsel an eine andere Einrichtungsstruktur. Der Begriff „Transition“ schließt sämtliche Abläufe und Veränderungen ein, die mit der Übergabe von Jugendlichen und jungen Erwachsenen mit chronischen und seltenen Erkrankungen von der Pädiatrie in die Erwachsenenversorgung einhergehen [3, 4].

## Relevanz des Themas

Durch den medizinischen Fortschritt und der damit einhergehenden steigenden Lebenserwartung erreichen viele Patientinnen und Patienten mit chronischen Erkrankungen, welche sich im Kindesalter manifestieren, das Erwachsenenalter. Diese sind auf eine lebenslange medizinische Betreuung, Versorgung und Beratung angewiesen. Sie haben im Jugendalter einen Transitionsbedarf, der eine wachsende Herausforderung für das Gesundheitssystem darstellt. Versorgungslücken während des Transitionsprozesses führen zu erhöhten Kosten für das Gesundheitssystem und stellen vor allem erhebliche Risiken für den Zustand der Betroffenen dar. Eine verminderte Lebensqualität sowie potenzielle Folgeschäden und Rückfälle in der Therapie sind dabei mögliche Konsequenzen [5]. Da die Zahl der Jugendlichen mit Transitionsbedarf weiter steigen wird, ist es von Bedeutung, übergreifende Standards für den Transitionsprozess anzustreben. Nur so kann langfristig eine gute medizinische Versorgung sichergestellt und ein reibungsloser Übergang in die Erwachsenenmedizin gewährleistet werden. Die Jugendlichen sollten frühzeitig ihrem Alters- und Entwicklungsstand entsprechend mit dem Thema Transition konfrontiert werden, um ihre Eigenständigkeit und Verantwortung im Umgang mit der Erkrankung zu fördern und eine gewisse Selbstständigkeit und Selbstverantwortung aufzubauen [6]. Der Transitionsprozess stellt eine bedeutende Herausforderung für das gesamte familiäre System dar, da die Verantwortung schrittweise von den Eltern den Jugendlichen übergeben wird. Zusätzlich müssen neue Fachärztinnen und Fachärzte für die fortlaufende Betreuung gefunden werden. Zu diesen Spezialistinnen und Spezialisten der Erwachsenenmedizin muss nun erstmals eine neue Beziehung aufgebaut werden, während zeitgleich langjährige und vertraute Beziehungen zur Pädiatrie beendet werden. Häufig verläuft dieser Prozess für Jugendliche nur unzureichend oder verzögert, was zu einem erhöhten Betreuungsbedarf in der Transitionsphase führt, der über die reguläre medizinische Versorgung hinausgeht und sowohl die Jugendlichen selbst als auch deren Familie betrifft [7, 8].

## Methodik

Im Rahmen einer Querschnittstudie wurden alle Patientinnen und Patienten der Universitätsklinik Innsbruck für Pädiatrie I (Allgemeine Pädiatrie) und III (Kardiologie und Pulmonologie) erfasst, die im Jahr 2023 mit Stand 31. Dezember 2023 in Betreuung waren. Dabei wurden folgende Bereiche berücksichtigt: Gastroenterologie und Hepatologie, Hämatologie und Onkologie, Nephrologie, Endokrinologie, Diabetologie, Rheumatologie, Neuropädiatrie, angeborene Stoffwechselstörungen, Pneumologie und Allergologie, Kardiologie und Cystische Fibrose. Die Pädiatrie II (Neonatologie) wurde in der Erhebung nicht berücksichtigt, da es keine Relevanz zum Thema Transition gibt. Die Erfassung der Gesamtanzahl der Patientinnen und Patienten in den einzelnen pädiatrischen Spezialbereichen erfolgte aus dem Klinik-Informationssystem (KIS). Es wurden alle Patientinnen und Patienten erfasst, die im Jahr 2023 an der Klinik für Pädiatrie betreut wurden. Anschließend wurde für jeden Spezialbereich die Gesamtanzahl ermittelt sowie die Altersgruppen 16 bis 18 Jahre und über 18 Jahre und in einer weiteren Tabelle gegenübergestellt. Um zu erheben, ob es sich bei den Patientinnen und Patienten über 18 Jahre um einen verzögerten Transfer oder um Patientinnen und Patienten handelt, die dauerhaft an der Pädiatrie betreut werden, wurden die Leiterinnen und Leiter der Spezialbereiche bzw. Ansprechpersonen befragt bzw. anhand der ID-Nummer der Betroffenen überprüft, ob diese im Jahr 2024 noch an der Pädiatrie in Betreuung gewesen waren oder bereits transferiert wurden.

Zusätzlich wurden anhand von Berichten aus Interviews mit den Ansprechpersonen (Leiterinnen und Leiter der Spezialbereiche oder Stellvertretende) Herausforderungen der Transition im Spezialbereich erfasst und ggf. Ansatzpunkte für eine verbesserte Transition erarbeitet.

## Ergebnisse

### Epidemiologische Daten

**Abb. 1:**
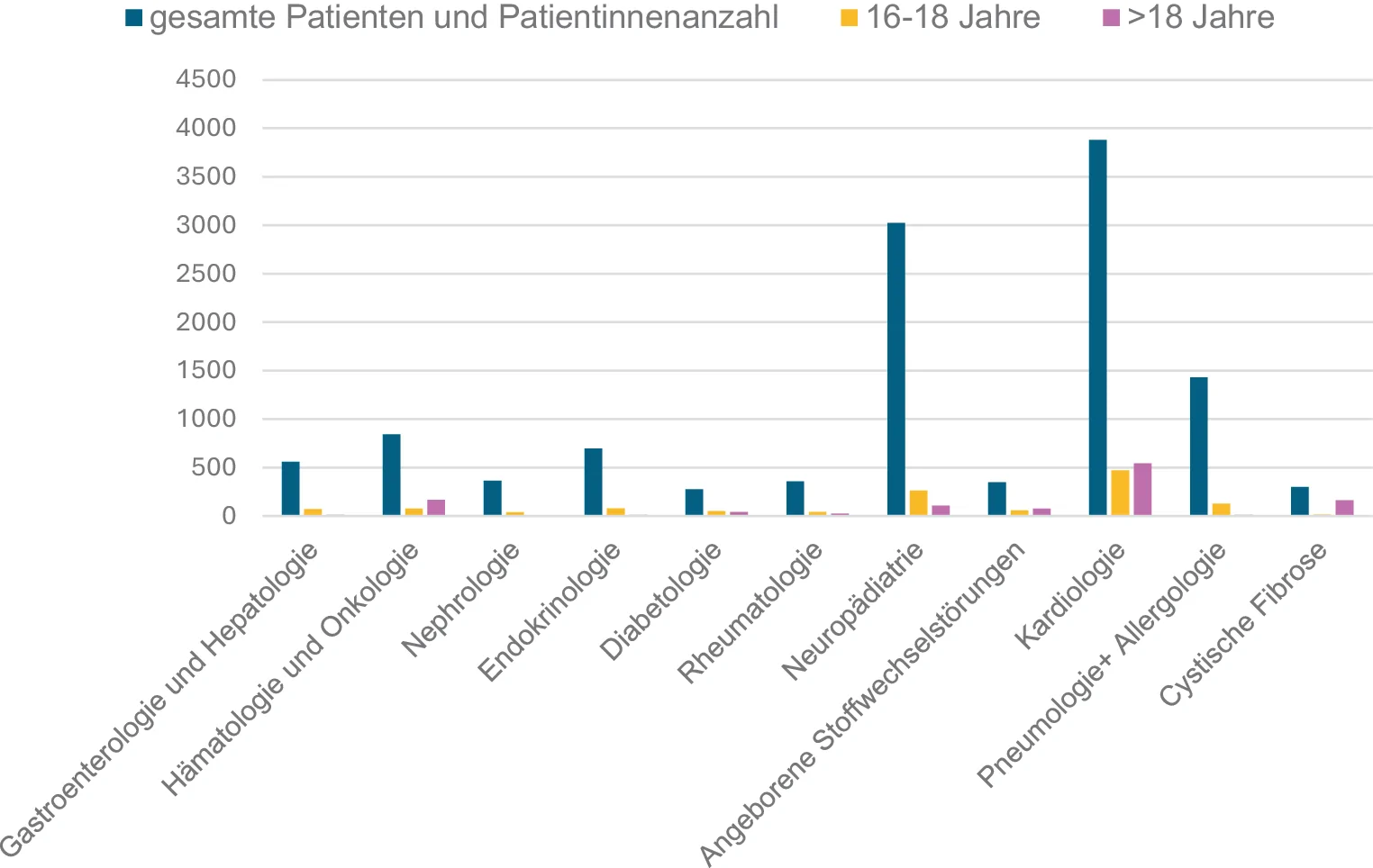
Säulendiagramm, Patientinnen- und Patientienanzahl mit ambulanter Vorstellung an der Pädiatrie I und III im Jahr 2023, Stand 31.12.2023, Gesamtanzahl ist in *blau* markiert, Patientinnen- und Patienten zwischen 16 und 18 Jahren in *gelb* und Patientinnen und Patienten über 18 Jahren in* pink*

Mit Stand 31. Dezember 2023 zählt die Pädiatrie I und III für das Jahr 2023 insgesamt 12.078 Patientinnen- und Patienteneinträge. Davon waren 6463 an der Pädiatrie I und 5615 an der Pädiatrie III vorstellig. Von den 6463 Patientinnen und Patienten der Pädiatrie I waren 677 zwischen 16 und 18 Jahren und 436 über 18 Jahre alt. Von den 5615 Patientinnen und Patienten der Pädiatrie III waren 611 zwischen 16 und 18 Jahren und 719 über 18 Jahre alt. Insgesamt waren von 12.078 Patientinnen und Patienten 1288 zwischen 16 und 18 Jahren und 1159 über 18 Jahre alt. Der jeweilige prozentuale Anteil der über 18 Jahre alten Patientinnen und Patienten ist für die einzelnen pädiatrischen Bereiche unterschiedlich (siehe Tab. 1 und Abb. 1).

**Tab. 1:** Patientinnen- und Patientenanzahl mit ambulanter Vorstellung an der Pädiatrie I und III im Jahr 2023, Stand 31.12.2023 aufgeteilt auf die pädiatrischen Spezialbereiche; Spalte 1 = Gesamtanzahl, Spalte 2 = Patientinnen und Patienten zwischen 16 und 18 Jahren, Spalte 3 = Patientinnen und Patienten über 18 Jahre, Spalte 4 = prozentueller Anteil der Patientinnen und Patienten über 18 Jahre, Spalte 5 = Altersspanne der über 18 Jährigen Patientinnen und Patienten, Spalte 6 = Median der über 18 Jährigen.

Pädiatrie I	Gesamt	16–18a	> 18a	Anteil > 18a (%)	Range > 18a	Median > 18a
Gastroenterologie und Hepatologie	558	71	11	1,97	18–31	18
Hämatologie und Onkologie	844	76	166	19,67	18–42	22
Nephrologie	365	39	5	1,37	18–18	18
Endokrinologie	697	78	11	1,58	18–44	18
Diabetologie	275	51	41	14,91	18–20	18
Rheumatologie	357	43	23	6,44	18–36	18,5
Neuropädiatrie	3023	261	107	3,54	18–64	33
Angeborene Stoffwechselstörungen	348	58	76	21,83	18–59	32,5
*Pädiatrie III*						
Kardiologie	3883	470	544	14,01	18–89	30
Pneumologie und Allergologie	1431	127	11	0,77	18–18	18
Cystische Fibrose	297	14	160	53,87	18–70	33

### Patientinnengruppe/Patientengruppe über 18 Jahre

In jedem Spezialbereich ist jeweils die Anzahl der 16–18 und > 18-Jährigen sowie der prozentuelle Anteil der über 18-Jährigen angeführt. Die Patientinnen- und Patientengruppe über 18 Jahre ist dabei die, bei der bereits der Transfer in die Erwachsenenmedizin erfolgt sein sollte. Bei den Bereichen Gastroenterologie und Hepatologie, Nephrologie, Endokrinologie, Diabetologie, Rheumatologie, Pneumologie und Allergologie ist diese erfolgt, es werden zwar noch ein paar wenige Prozent über 18-Jährige erfasst, allerdings zeigt der Median von 18 bis 18,5 Jahren, dass die Transition bevorsteht, und meist aus logistischen Gründen (gerade 18 geworden, Termin an Erwachsenenmedizin vereinbart, aber noch nicht stattgefunden) noch nicht vollzogen ist.

Im Bereich z. B. *Diabetologie* sind 41 (14,91 %) der Patientinnen und Patienten über 18 Jahre alt. Von diesen 41 Betroffenen sind 23 Patientinnen und Patienten gerade 18 Jahre, 16 sind 19 Jahre und 2 sind 20 Jahre alt. Ein zeitnaher Transfer dieser Patientinnen und Patienten wird angestrebt.

In den anderen Bereichen, in denen die Transition erfolgt ist, liegt der Anteil der über 18-Jährigen unter 10 %.

In der *Rheumatologie* sind 23 (6,44 %) Patientinnen und Patienten über 18 Jahre. Von diesen 23 Patientinnen und Patienten waren 7 gerade 18 Jahre und wurden kurz anschließend transferiert, 3 Erwachsene wurden nur einmal im Rahmen einer Familienuntersuchung vorstellig, bei 2 wurde die Behandlung mit 19 Jahren abgeschlossen, und 11 weitere befinden sich noch in Behandlung, wobei alle unter 20 Jahre alt sind und individuell zeitnah transferiert werden.

In der *Neuropädiatrie* sind 107 (3,54 %) Patientinnen und Patienten über 18 Jahre. Dabei handelt es sich vor allem um Patientinnen und Patienten mit syndromalen Erkrankungen, die mehrere Organsysteme betreffen und mit einer geistigen Behinderung einhergehen, für die (noch) keine geeignete Anlaufstelle an der Erwachsenenmedizin gefunden wurde.

In der *Gastroenterologie und Hepatologie* sind 11 (1,97 %) Patientinnen und Patienten über 18 Jahre. Von diesen 11 wurden fünf mit 18 Jahren an die Erwachsenenmedizin transferiert, zwei waren zu einem einmaligen Konsil vorstellig und vier benötigten keine Transition, da sie aufgrund der bei ihnen vorliegenden Erkrankung (Lebertransplantation, entzündlicher Pseudotumor, Kurzdarmsyndrom und Chloriddiarrhoe) an der Pädiatrie in Betreuung bleiben. Diese vier Betroffenen haben ein Alter von 18,18,19 und 31 Jahren.

In der *Endokrinologie *sind 11 (1,58 %) Patientinnen und Patienten über 18 Jahre. Von diesen 11 Betroffenen wurden neun kurz nach ihrem 18. Geburtstag transferiert und zwei befinden sich noch in Behandlung (42 und 44 Jahre alt) (genetischer Kleinwuchs, CPOX-Defizienz).

In der *Nephrologie* sind fünf (1,37 %) Patientinnen und Patienten über 18 Jahre. Davon sind alle fünf gerade erst 18 Jahre alt geworden. Von diesen fünf 18-Jährigen wurde einer austherapiert, drei wurden im selben Jahr transferiert und ein weiterer steht kurz vor der Transferierung und ist mit 18 Jahren wegen einer Nierendysplasie noch an der Nephrologie in Behandlung.

Die *Pneumologie und Allergologie* ist der Bereich mit dem kleinsten prozentuellen Anteil der Patientinnen und Patienten über 18 Jahre. Dort sind 11 Patientinnen und Patienten (0,77 %) über 18 Jahre. Diese 11 wurden alle mit 18 Jahren transferiert.

Die Bereiche Cystische Fibrose, Hämatologie & Onkologie, Kardiologie und Angeborene Stoffwechselstörungen zeigen hingegen Besonderheiten, was den Transfer an die Erwachsenenmedizin betrifft.

Der Bereich *Cystische Fibrose (CF)* ist der mit den meisten Patientinnen und Patienten über 18 Jahre. In diesem Spezialbereich sind 53,87 % (*n* = 160) Patientinnen und Patienten über 18 Jahre. Vereinbarungsgemäß werden CF Patientinnen und Patienten in der pädiatrischen CF Ambulanz weiterbetreut, wobei innerhalb der pädiatrischen Ambulanz ein klarer Transitionsprozess definiert ist. Da CF Patientinnen und Patienten laut Leitlinien in entsprechenden Zentren betreut werden sollen und bisher keine Möglichkeit auf der Inneren Medizin besteht, bleiben sie vereinbarungsgemäß langfristig an der CF Ambulanz der Klinik für Pädiatrie in Betreuung.

Ähnlich wie im Bereich Cystische Fibrose ist es in der *Hämatologie und Onkologie, *in der 19,67 % (*n* = 166) der Patientinnen und Patienten über 18 Jahre alt sind. In diesem Bereich werden die Patientinnen und Patienten seit 2021 durch das Programm „ZONE“ (= Zentrum für onkologische Nachsorge für junge Erwachsene) weiterbetreut. Dieses Programm übernimmt die Funktion der Transition, jedoch auch die Langzeitnachsorge der Patientinnen und Patienten. Die Betroffenen bleiben an der Klinik angebunden, da es eine Indikation für eine Langzeitnachsorge für onkologische Patientinnen und Patienten laut aktuellen Leitlinien gibt. Aktuell wird das Programm ZONE durch Pädiaterinnen und Pädiater geleitet. Eine Externalisierung aus der Kinderklinik sowie eine gemeinsame Betreuung mit der Inneren Medizin der Erwachsenenmedizin wird in Kürze umgesetzt. Dadurch kann die Transition und die Langzeitnachsorge komplett außerhalb der Kinderklinik von Seiten der Pädiaterinnen und Pädiater eingeleitet und weiterführend gemeinsam mit den Internistinnen und Internisten durchgeführt werden. Aktuell ist das mediane Alter mit 22 Jahren (Range 18–42) im Bereich Hämatologie und Onkologie deutlich niedriger als das im Bereich Cystische Fibrose mit 33 Jahren (18–70).

In der *Kardiologie* ist der Anteil an Patientinnen und Patienten über 18 Jahre 14,01 % (*n* = 544). Dabei handelt es sich Großteils um Betroffene mit angeborenen Herzfehlern bzw. chronischen seltenen Erkrankungen (Marfan-Syndrom), welche nicht transferiert werden können, da es keine passende Anlaufstelle in der Erwachsenenmedizin gibt.

Der Bereich mit dem zweitgrößten prozentualen Anteil von Patientinnen und Patienten über 18 Jahre ist der Bereich der *Angeborenen Stoffwechselstörungen* mit 21,83 % (*n* = 76). Von diesem Bereich konnten bisher z. T. aus Kapazitätsgründen von Seiten der Erwachsenenmedizin nur Patientinnen und Patienten mit den drei Krankheitsbildern Phenylketonurie, Propionazidämie und Morbus Fabry transferiert werden. Für manche andere Krankheitsbilder wird noch nach entsprechenden Ressourcen gesucht. Da es sich oft um Multisystemerkrankungen handelt, ist es nicht ganz einfach, die geeignete Anlaufstelle dafür zu finden.

### Herausforderungen des Transitionsprozesses

Die Interviews zum Thema Transition mit den jeweiligen Ansprechpersonen zeigten unterschiedliche Problematiken des Transitionsprozesses. Gewisse Gemeinsamkeiten gibt es dennoch in allen Fachbereichen.

Eine beständige *Herausforderung* ist das oft vorliegende mangelnde Selbstmanagement bzw. eine unzureichende Selbstständigkeit der Patientinnen und Patienten mit chronischen Erkrankungen. Außerdem besteht im Jugendalter auch häufig das Problem der Krankheitseinsicht – auch wenn sich die Betroffenen der Krankheit bewusst sind, wird diese oft verdrängt und andere – für dieses Lebensalter wichtigere – Prioritäten gesetzt. Eine weitere Herausforderung ist das überlastete Gesundheitssystem, d. h. fehlende, vor allem zeitliche und personelle Ressourcen, die den Transitionsprozess erschweren. Der Transitionsprozess wird sowohl von medizinischen sowie gesellschaftlichen Faktoren beeinflusst.

Es gibt unterschiedliche *Verbesserungsansätze* für den Transitionsprozess, um eine sichere und kontinuierliche Versorgung zu gewährleisten. Einerseits muss das medizinische Personal sowohl der Kinder und Jugendmedizin wie auch der Erwachsenenmedizin gezielt auf den Transitionsprozess geschult werden. Nur dadurch kann den Patientinnen und Patienten mit chronischen Erkrankungen eine passende Anlaufstelle angeboten werden für eine sichere Versorgung über das 18. Lebensjahr hinaus. Andererseits wird der Transitionsprozess positiv beeinflusst, wenn er stufenweise erfolgt, indem der Patientin oder dem Patienten genügend Zeit zum Loslassen von Seiten der Pädiatrie gegeben wird, jedoch auch ausreichend Zeit zum Ankommen in der Erwachsenenmedizin besteht. Nicht nur die Betroffenen, auch die Eltern, Geschwister und die erweiterte Familie, müssen stufenweise in einem strukturierten, standardisierten Prozess auf die Transition vorbereitet werden. Schließlich sind Absprachen und Austausch zwischen den Pädiaterinnen und Pädiatern mit den Internistinnen und Internisten und ein gegenseitiges Feedback wichtig. Eine enge Zusammenarbeit und ein beidseitiges Interesse tragen dazu bei, auf wichtige, individuelle und patienteninnen- und patientenbezogene Erkenntnisse Rücksicht zu nehmen. Es ist essenziell Ressourcen wie Zeit und Personal bereitzustellen, um weitere Unterstützungsangebote zu schaffen. All dies sind Voraussetzungen für eine gelungene Transition.

### Limitierende Faktoren

Ein limitierender Faktor der Studie ist die Zuordnung der Patientinnen und Patienten zum jeweiligen Spezialbereich. Es kann nicht ausgeschlossen werden, dass vereinzelt Patientinnen und Patienten nicht korrekt in die Spezialbereiche kodiert wurden und so nicht in der „richtigen“ Kohorte erfasst wurden. Es kann auch sein, dass Patientinnen und Patienten nur stationär behandelt wurden und daher in der Ambulanzstatistik nicht aufscheinen, oder dass Patientinnen und Patienten seltener als einmal pro Jahr zur ambulanten Vorstellung kommen und daher im Jahr 2023 nicht erfasst wurden. Zudem handelt es sich um Single-Center-Daten eines universitären Zentrums, die spezifische strukturelle und organisatorische Gegebenheiten widerspiegeln, wodurch die Vergleichbarkeit mit anderen Regionen nur eingeschränkt gegeben ist. Bei einer Anzahl von über 12.000 Patientinnen und Patienten dürfte sich das Gesamtbild dadurch aber nicht wesentlich ändern.

Von den Interviews ist zu sagen, dass es sich bei den Antworten um persönliche Meinungen und Wahrnehmungen der Ansprechperson des jeweiligen Spezialbereichs handelt, so dass es zu Verzerrungen kommen kann, sowohl in die positive, als auch in die negative Richtung. Insgesamt lässt sich allerdings aus den Interviews ein Gesamtbild ableiten.

## Diskussion

Die Ergebnisse der Querschnittstudie sowie der Interviews verdeutlichen, dass Transition und Transfer in den einzelnen Fachbereichen der Pädiatrie Innsbruck unterschiedlich gut funktionieren und dabei verschiedene Transitionsarten bestehen. Während man in den Bereichen, bei denen die Erkrankungen bereits auch bei Erwachsenen betreut werden, z. B. Gastroenterologie und Hepatologie, Nephrologie, Endokrinologie, Diabetologie, Rheumatologie, Neurologie und Pneumologie und Allergologie, eine gut erfolgte Transition sieht, ist das bei Bereichen, bei denen die Erkrankungen z. T. bei Erwachsenen nicht bekannt sind oder die mehrere Organsysteme betreffen, z. B. Angeborene Stoffwechselstörungen und Kardiologie, nur teilweise möglich. In den Bereichen Hämatologie und Onkologie, sowie Cystische Fibrose findet in unserer Klinik vereinbarungsgemäß kein Transfer statt, da die Patientinnen und Patienten ohnehin auf eine Versorgung im Zentrum angewiesen sind.

Im Vergleich zur Universitätsklinik Innsbruck liefert die bundesweite deutsche Studie „Transition for adolescents with a rare disease: results of a nationwide German project“ wichtige Hinweise auf den Ablauf und die Erfolgsfaktoren von Transitionen bei seltenen Erkrankungen. In dieser Studie wurden 292 Patientinnen und Patienten in einen strukturierten Transition-Pfad aufgenommen, von denen 286 den gesamten Prozess abgeschlossen haben. Bemerkenswert ist, dass in 267 Fällen der Transfer in die Erwachsenenmedizin erfolgreich umgesetzt werden konnte, während nur bei wenigen Betroffenen eine weitere pädiatrische Betreuung notwendig war, weil keine geeignete Anlaufstelle in der Erwachsenenmedizin verfügbar war. Die deutschen Ergebnisse zeigen, dass eine standardisierte und koordinierte Betreuung, bei der Übergabeprotokolle und interdisziplinäre Übergabegespräche verpflichtend sind, die Adhärenz, kontinuierliche Versorgung und letztlich auch die Lebensqualität der Jugendlichen deutlich verbessern kann. Insbesondere die frühzeitige Einbindung der Patienten in den Transition-Prozess sowie die enge Zusammenarbeit zwischen Pädiatrie und Erwachsenenmedizin trugen wesentlich zum Erfolg bei [9].

Auch andere österreichische Kliniken (Graz, Salzburg, Wien) bemühen sich, seit einigen Jahren um den Aufbau von Transitionsmodellen gemäß der 2021 veröffentlichenten AWMF S3-Leitlinie der Gesellschaft für Transitionsmedizin: Leitlinie Transition von der Pädiatrie in die Erwachsenenmedizin [6]. Die Transition gelingt dort gemäß den aufgebauten Strukturen zwischen der Pädiatrie und der Erwachsenenmedizin in etwa entsprechend der Innsbrucker Situation, mancherorts etwas besser, z. B. in Wien, wo an der Endokrinologie der Inneren Medizin alle Patientinnen und Patienten mit Angeborenen Stoffwechselstörungen über 18 Jahren betreut werden.

Auch in der S3-Leitlinie der Gesellschaft für Transitionsmedizin werden Transitionsfördernde und -erschwerende Faktoren identifiziert ([6, 10–14]; siehe Tab. 2).

**Tab. 2:** Transitionsfördernde und -erschwerende Faktoren (aus [6, 10–14]).

Transitionsfördernde Faktoren	Transitionserschwerende Faktoren
Frühzeitige,schrittweise, altersentsprechende Vorbereitung der gesamten beteiligten Personen	Persönliche Gründe: mangelndes Interesse, andere Prioritäten, vor allem im Jugendalter, geringes Krankheitsgefühl, zu wenig Aufklärung und Wissen über die Erkrankung
Bestehender Kontakt zur weiterbetreuenden Anlaufstelle	Nicht ausreichender Austausch zwischen den Betreuungssystemen
Enger Kontakt und Absprache zwischen der Pädiatrie und der Erwachsenenmedizin	geringer, ausbaufähiger Austausch
Erkrankungen, die nur ein Organsystem betreffen	Komplexe Erkrankungen, die mehrere Organsysteme betreffen oder mit geistiger und körperlicher Beeinträchtigung und daher höherem Betreuungsaufwand (syndromale Erkrankungen)
Therapien, die auch in der Erwachsenenmedizin identisch fortgeführt werden können (z. B. Insulin Therapie)	Fehlende Expertise in der Erwachsenenmedizin
Erkrankungen mit höherer Inzidenz	Seltene Erkrankungen

Die Chancen auf eine höhere Lebensqualität und Lebenserwartung sind bei gelungener Transition durch die Grunderkrankung sehr individuell. Medizinisch gesehen haben Patientinnen und Patienten mit gelungener Transition bessere Laborparameter, eine konstantere Betreuung und eine bessere Therapieadhärenz, was sich in einer geringeren Anzahl von Hospitalisierungen widerspiegelt [10]. Patientinnen und Patienten, die von Seiten der Pädiatrie entlassen wurden und von einem medizinischem Team der Erwachsenenmedizin weiterbetreut wurden, ohne dass der/die Arzt/Ärztin wechselt, zeigten ein 77 % geringeres Risiko einer Rehospitalisierung im Vergleich zu Patientinnen und Patienten, welche zu einem neuen Arzt oder zu einer neuen Ärztin überwiesen wurden [13]. Des Weiteren zeigen Patientinnen und Patienten bei gelungener Transition weniger Akutgeschehen und weniger Langzeitfolgen im Verlauf. Eine gelungene Transition beeinflusst neben den medizinischen Faktoren das Privatleben positiv. Dadurch kann das Bildungs- und Ausbildungsniveau gesteigert werden, was wiederum die Integration in die Gesellschaft und die Selbstständigkeit fördert [12, 15, 16].

## Conclusio

Die Querschnitt-Studie an unserem Zentrum zeigt, dass in einigen Bereichen eine gelungene Transition, in anderen noch Verbesserungspotential besteht.

Da eine erfolgreiche Transition ein wesentlicher Faktor in der Morbidität und auch Mortalität von Jugendlichen mit chronischen und seltenen Erkrankungen ist, ist es von gesellschaftlich- und gesundheitspolitischem Interesse, die Strukturen rund um dieses Thema gemäß der S3 Leitlinie: Transition von der Pädiatrie in die Erwachsenenmedizin [6] flächendeckend umzusetzen.

Dabei ist eine möglichst vollständige Übergabe der medizinischen Vorgeschichte ohne Informationsverlust und ohne plötzliche Unterbrechung der notwendigen medizinischen Betreuung wichtiger als ein pünktlicher Transfer in die Erwachsenenmedizin zum 18. Geburtstag, da diese Risikofaktoren für eine schlechtere Adhärenz und schlechteren Krankheitsverlauf darstellen [17].
